# Use of the structure-function relationship in detecting glaucoma progression in early glaucoma

**DOI:** 10.1186/1471-2415-14-118

**Published:** 2014-10-04

**Authors:** Kazuyuki Hirooka, Saki Manabe, Kaori Tenkumo, Eri Nitta, Shino Sato, Akitaka Tsujikawa

**Affiliations:** Department of Ophthalmology, Kagawa University Faculty of Medicine, 1750–1 Ikenobe, Miki, Kagawa, 761-0793 Japan

**Keywords:** Optical coherence tomography, Visual field, Retinal nerve fiber layer, Glaucoma progression

## Abstract

**Background:**

To evaluate the use of optical coherence tomography (OCT) retinal nerve fiber layer (RNFL) thickness and visual field (VF) measurements in detecting disease progression in patients with early glaucoma.

**Methods:**

Over a 3-year period, this study examined 60 eyes of 39 glaucoma patients whose total deviation in the superior or inferior hemifield was more than -6 dB. All eyes underwent at least four serial RNFL measurements performed by Cirrus OCT, with the first and last measurements separated by at least three years. On the same day as the RNFL imaging, VF testing was also performed by using the Swedish Interactive Threshold Algorithm Standard 30–2 program of the Humphrey Field Analyzer. Serial RNFL thicknesses and VF progression were assessed using the Guided Progression Analysis (GPA) software program. RNFL thickness progression and VF progression were evaluated by the event analysis.

**Results:**

The mean observation period was 57.6 ± 10.0 months, and during this time, a total of 366 OCT and 366 VF measurements were performed. Using only OCT, progression was found in 2 eyes, while progression was found in 1 eye when only using VF GPA. When combined measurement findings were used, the analysis found progression in 8 eyes.

**Conclusions:**

When mild VF defect is present, OCT RNFL thickness measurements can be helpful in discerning glaucoma progression.

## Background

Glaucoma is a progressive optic neuropathy characterized by the loss of retinal ganglion cells and the retinal nerve fiber layer (RNFL), with an associated visual field loss [1, 2]. In glaucoma, the precise nature of the relationship between the structure and function is important, as it can be used for detecting glaucomatous damage, determining the stage of the disease, and monitoring the progression of the disease.

In routine clinics, spectral-domain optical coherence tomography (SD-OCT) has rapidly become one of the most widely used technologies due to its high image resolution and measurement precision. Changes in the appearance of the RNFL thickness often precede the development of glaucomatous visual field (VF) loss [3]. As a way of improving our ability to detect the presence and progression of glaucomatous damage, a number of studies have used SD-OCT to specifically focus on the relationship between the structural and functional damage. Results of these studies have demonstrated there are high correlations between the global VF sensitivity and the peripapillary RNFL [4, 5]. In contrast, however, several other studies have reported finding poor agreement between the structure- and function-based results and the extent of the progression [6–8]. We have recently reported that the average of the progressive hemifield total deviation (TD) at baseline for the both RNFL and VF progression was -3.21 ± 1.38 dB, while individually it was -2.17 ± 1.14 dB for RNFL progression and -9.12 ± 3.75 dB for VF progression [9]. In this study, if progression was noted in the inferior RNFL thickness, we then evaluated the TD of the superior hemifield. The average of the progressive hemifield TD demonstrated that there was a significant advancement of VF progression as compared to RNFL progression. Based on these results, we speculated that determination of the RNFL thickness by OCT might be an important diagnostic tool, as these measurements should be able to help discern glaucoma progression when the VF defect is mild.

Therefore, the purpose of our current study was to examine the relationship between the RNFL thickness and VF measurements, and then evaluate the ability of using these findings to determine progression in patients with early glaucoma.

## Methods

### Patients

We performed a retrospective review of patients with glaucoma who underwent OCT measurements of RNFL thickness and VF testing at Kagawa University Hospital, Kagawa, Japan, between October 2008 and January 2014. Each of the eyes underwent at least four serial RNFL measurements, with the first and last measurements separated by at least 3 years. All eligible subjects received a detailed explanation of the study and signed an informed consent form in accordance with the principles embodied in the Declaration of Helsinki. This study was approved by the Institutional Review Board at Kagawa University Hospital. At the baseline examination, all subjects underwent a complete ophthalmic evaluation that included visual acuity testing with refraction, intraocular pressure, and dilated fundus examination with stereoscopic biomicroscopy of the optic nerve head using slit-lamp and indirect ophthalmoscopy. To be included in the study, all subjects had to have a best-corrected visual acuity of 20/40 or better, a spherical error within a range between +4.0 and -6.0 diopters, and a cylinder within ± 2.0 diopters. Exclusion criteria included a history of any kind of retinal pathology, retinal laser procedure, retinal surgery, or neurologic disease. In addition, we also excluded patients with advanced hemifield VF defect who had an initial TD in the superior or inferior hemifield under -6 dB. However, if the superior and the inferior hemifield were each above -6 dB, we included both hemifields. In addition, since we assessed each hemifield in these patients, the global TD was not included in the exclusion criteria. Glaucomatous eyes were defined as eyes exhibiting structural glaucomatous changes (vertical cup-disc asymmetry between fellow eyes of ≥ 0.2, a cup-disc ratio of ≥ 0.6, and a neuroretinal rim narrowing, notches, localized pallor, or RNFL defects with glaucomatous VF loss in the corresponding hemifield). A glaucomatous VF was defined as a glaucoma hemifield test outside of the normal limits on at least two consecutive baseline tests and the presence of at least three contiguous test points within the same hemifield on the pattern deviation plot at *P* < 1%, with at least one at *P* < 0.5% (excluding those points on the edge of the field or those directly above and below the blind spot). VF testing and RNFL imaging were performed during the same visit.

### Cirrus HD-OCT RNFL measurement

Cirrus HD-OCT uses spectral domain technology (Carl Zeiss Meditec, Dublin, CA). This technique utilizes an optic disc cube that is generated from a 3-dimensional data set composed of 200 A-scans from each of 200 B-scans that cover a 6 mm^2^ area centered on the optic disc. After creating an RNFL thickness map from the cube, the software automatically determines the center of the disc and then extracts a circumpapillary circle (1.73 mm radius) from the data set. All of the images obtained in the current study had signal strengths of at least 6. The RNFL thickness deviation and the RNFL thickness change maps were automatically generated by the OCT instrument and exported to a computer that analyzed the progression pattern of the RNFL defects. The RNFL defects were visualized in the RNFL thickness deviation map, which was composed of 50 × 50 pixels. A pixel was coded in yellow if the RNFL measurement was below the lower 95% and coded in red if below 99% of the percentile ranges for that particular pixel.

The RNFL thickness change map was a component of the Guided Progression Analysis (GPA) software (Carl Zeiss Meditec), which provided both event- and trend-based analysis of the RNFL progression based on the serial RNFL thickness maps. This software automatically aligned and registered the baseline and follow-up OCT images so that the same pixel locations could be measured for change. In order to be able to generate a GPA report, at least four patient visits were necessary. The GPA program then overlaid and compared the serial RNFL thickness against the duration of the follow-up.

### Visual field examination

Standard visual field testing was performed using static automated white-on-white threshold perimetry (Humphrey Field Analyzer II (HFA); Carl Zeiss Meditec, Dublin, CA), using the 30–2 Swedish Interactive Threshold Algorithm Standard test. The visual field was defined as reliable only when the fixation losses and the false-positive and false-negative rates were less than 20%. Only reliable test data were used in all of our analyses.

The GPA software compared the patient’s baseline visual field to each of the subsequent visual fields determined during the follow-up examinations. All baseline values were obtained by averaging the data from the first two exams. The progression evaluation was performed relative to the baseline. The evaluation of the progression was carried out by comparing the threshold modifications to a database of stable glaucoma patients who were tested over a very short period of time. These evaluations took into account the fluctuations related to eccentricity and advancing disease. Each of the consecutive tests examined the same locations (≥3) and determined if there was progression during any of the consecutive VF tests. Based on the findings of a previous study [10], “possible progression” was considered to be present when the GPA printouts reported finding progression in 2 locations, while “likely progression” was considered to be present when progression was found in 3 locations.

### Analysis

Using the GPA software, RNFL thickness progression was assessed by event analysis. Event analysis defined progression as a change that exceeded a predefined limit when compared to the baseline value. The baseline values were obtained by averaging the data from the first two exams. After completing a series of RNFL thickness measurements, the GPA software then compared the baseline RNFL thickness value to the final measurement value. A “possible loss” was identified when the differences between the baseline and final measurement values exceeded the test-retest variability. If the “possible loss” criterion was met on two successive visits, the patient was considered to show a “likely loss”. In the current study, if the RNFL Thickness Map Progression indicated that there was a “likely loss” or “possible loss”, we defined the RNFL thickness as having progressed. The VF GPA classification indicated “likely progression” or “possible progression”, this was defined as progression of the glaucoma.

### Statistics

All statistical values are presented as mean ± standard deviation (SD), with *P* values < 0.05 considered statistically significant. Data were analyzed using a paired *t*-test. Statistical analyses were performed using SPSS version 19.0 (IBM, New York).

## Results

We analyzed 366 OCT scans and 366 VF test results obtained from 60 eyes of 39 patients with glaucoma (Table 1). The average number of OCT scans and VF tests for each eye was 5.4. The follow-up duration ranged from 36 to 70 months. Table 1 presents the patient’s demographics, along with the VF and RNFL measurements. Mean age was 64.9 ± 10.9 years. The baseline visual field TD and RNFL thickness values were -2.75 ± 1.40 dB (range, -0.79 to -6.00 dB) and 90.6 ± 20.6 μm (range, 45 to 140 μm), respectively. Significant differences were observed between the baseline and the final TD and RNFL thickness measurements (*P* < 0.05).

**Figure 1:**
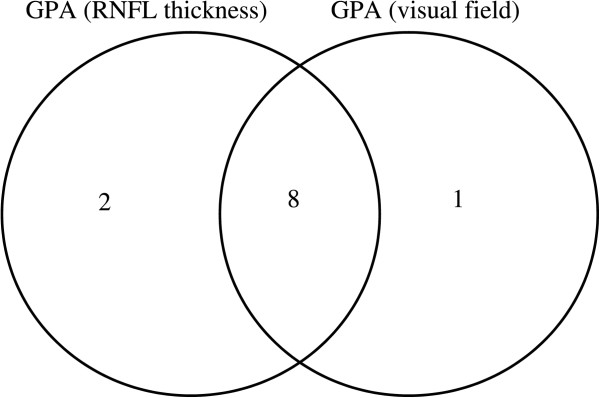
**A Venn diagram comparing the number of eyes with progression as determined by OCT GPA and by HFA GPA.** The numbers within the diagram indicate the number of subjects found with progression of the disease.

Eight eyes had concurrent progression in structure and function. Topographical correspondence between the VF progression and the RNFL progression was present in 8 eyes (Figure 1). In 3 of the 8 eyes, OCT identified damage before it was detected by HFA. In 4 of the 8 eyes, both OCT and HFA detected progression at the same time. In 1 out of 8 eyes, HFA identified progression before it was detected by OCT. OCT GPA reported that 2 eyes exhibited progression, while VF GPA showed there was progression in 1 eye (Figure 1).

**Table 1:** **Demographics, visual field, and OCT RNFL measurements**

Demographics	
Subjects	43
Eyes	68
Gender (male/female)	17/26
Age (y)	64.9 ± 10.9
Visual field measurements	
Visual field tests	356
Average visual field tests per eye	5.4 ± 0.9
Baseline TD (dB)	-2.75 ± 1.40
TD at final visit (dB)	-4.05 ± 2.89*
OCT-RNFL measurements	
OCT tests	356
Average OCT tests per eye	5.4 ± 0.9
Baseline average RNFL thickness (μm)	90.6 ± 20.6
Average RNFL thickness at final visit (μm)	87.7 ± 20.8**

*P < 0.001 compared with baseline.

**P < 0.05 compared with baseline.

TD, total deviation; dB; decibels, OCT, optical coherence tomography RNFL, retinal nerve fiber layer.

**Figure 2:**
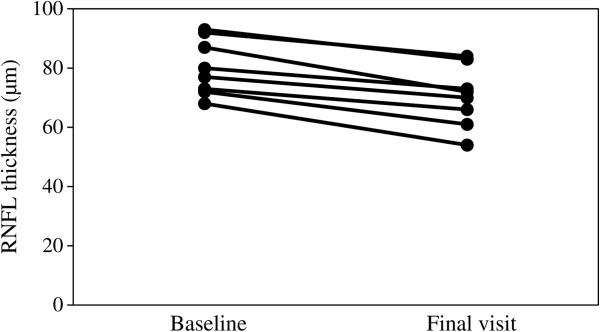
**RNFL thickness changes in the 8 patients who fit progression criteria by OCT and HFA.**

Table 2 shows the RNFL measurements in eyes with or without progression. Significant differences were observed between the baseline and the final measurements (*P* < 0.001). Figure 2 shows the initial and final RNFL thickness in each eye with progression.

**Table 2:** **RNFL measurements in eyes with or without progression**

	Eyes with progression	Eyes without progression
	(8 eyes)	(52 eyes)
Baseline average RNFL thickness (μm)	83.6 ± 19.7	91.9 ± 20.6
Average RNFL thickness at final visit (μm)	72.9 ± 25.4*	90.6 ± 18.6

*P < 0.05 compared with baseline.

RNFL, retinal nerve fiber layer.

In 1 eye, VF GPA indicated there was significant improvement. However, OCT in this eye showed the opposite, indicating that there was no significant improvement.

## Discussion

Worsening optic disc excavation, RNFL atrophy and deterioration in visual function are all used to detect the progression of glaucoma. If progression is confirmed, treatment needs to be modified or enhanced in order to prevent further irreversible loss of the patient’s visual function. In the current study, we evaluated the effectiveness of using OCT RNFL thickness and VF measurements by HFA to detect progression in patients with early glaucoma.

It has been previously recommended that glaucoma be monitored by both VF and digital imaging devices [11]. However, it has been reported that there is poor agreement between the VF and RNFL results when used to detect progression [6–8]. Structural assessment by OCT has been shown to be much worse when performed during advanced stages of damage [12]. It has been further reported that a complete loss in standard automated perimetry sensitivity is not due to an RNFL thickness of zero, but is in fact is associated with a finite RNFL thickness. Previous findings have also shown that there is a minimum value beyond which the thickness cannot be reduced, as glial cells or other factors do not contribute to the number of axons [12]. For these reasons, our study excluded the initial TD in the superior or inferior hemifield when values were under -6 dB as changes in RNFL thickness with this advanced loss are minimal.

In 7 of the 8 eyes in which both techniques reported progression, OCT was able to identify damage prior to or at the same time that HFA detected the damage. Results of previous cross-sectional studies in early glaucoma patients have also suggested that progression determined by the RNFL thickness is more noticeable than that determined by the visual field, based on the observed curvilinear structure-function relationship [13–15]. Furthermore, it is notable that depending on the stage of the disease, the ability to detect progression may vary considerably between the structural and functional tests. HFA seems to be relatively insensitive when detecting changes in early glaucoma, whereas OCT seems to perform relatively worse during the advanced stage of glaucoma.

In our current study, an event-based approach was selected for analysis of both the structural and functional progression. When using trend analysis, one important assumption is that the amount of change will be linearly proportional to the duration of the follow-up. However, it is known that the rate of progression can vary over time due to loosening or tightening of the control of the glaucoma treatment, as well as due to changes related to the natural progression of the disease. For this reason, our study assessed the functional and structural progressions by using the event analysis of the GPA software for both the HFA and OCT.

The limitations of our present work include having a small sample size and a small number of tests. Therefore, larger studies will need to be undertaken in the future in order to be able to more precisely determine the structure-function relationship present when detecting the glaucoma progression.

## Conclusions

In conclusion, our current study demonstrated in this limited group that there was good agreement between the structural and functional tests used to evaluate glaucoma progression during the early stage of the disease.

## References

[1] Quigley HA, Addicks EM, Green WR (1982). Optic nerve damage in human glaucoma. III. Quantitative correlation of nerve fiber loss and visual field defect in glaucoma, ischemic neuropathy, papilledema, and toxic neuropathy. Arch Ophthalmol.

[2] Quigley HA, Dunkelberger GR, Green WR (1989). Retinal ganglion cell atrophy correlated with automated perimetry in human eyes with glaucoma. Am J Ophthalmol.

[3] Hood DC, Kardon RH (2007). A framework for comparing structural and functional measures of glaucomatous damage. Prog Retina Eye Res.

[4] Takagishi M, Hirooka K, Baba T, Mizote M, Shiraga F (2011). Comparison of retinal nerve fiber layer thickness measurements using time domain and spectral domain optical coherence tomography, and visual field sensitivity. J Glaucoma.

[5] Nilforushan N, Nassiri N, Moghimi S, Law SK, Giaconi J, Coleman AL, Caprioli J, Nouri-Mahdavi K (2012). Structure-function relationships between spectral-domain OCT and standard achromatic perimetry. Invest Ophthalmol Vis Sci.

[6] Wollstein G, Schuman JS, Price LL, Aydin A, Stark PC, Hertzmark E, Lai E, Ishikawa H, Mattox C, Fujimoto JG, Paunescu LA (2005). Optical coherence tomography longitudinal evaluation of retinal nerve fiber layer thickness in glaucoma. Arch Ophthalmol.

[7] Leung CK, Cheung CY, Weinreb RN, Qiu K, Liu S, Li H, Xu G, Fan N, Pang CP, Tse KK (2010). LamDS. Evaluation of retinal nerve fiber layer progression in glaucoma: a study on optical coherence tomography guided progression analysis. Invest Ophthalmol Vis Sci.

[8] Leung CK, Liu S, Weinreb RN, Lai G, Ye C, Cheung CY, Pang CP, Tes KK, Lam DS (2011). Evaluation of retinal nerve fiber layer progression in glaucoma: a prospective analysis with neurotetinal rim and visual field progression. Ophthalmology.

[9] Tenkumo K, Hirooka K, Baba T, Nitta E, Sato S, Shiraga F (2013). Evaluation of relationship between retinal nerve fiber layer thickness progression and visual field progression in patients with glaucoma. Jpn J Ophthalmol.

[10] Heijl A, Leske MC, Bengtsson B, Bengtsson B, Hussein M, Early Manifest Glaucoma Trial Group (2003). Measuring visual field progression in the Early Manifest Glaucoma Trial. Acta Ophthalmol Scand.

[11] Consensus statements: ** .** In *Glaucoma Diagnosis Structure and Function: Reports and Consensus Statements of the 1st Global AIGS Meeting on “Structure and Function in the Management of Glaucoma”*. Edited by: Weinreb RN, Greve EL. The Netherlands: Kugler: The Hague; 2004:155–156.

[12] Hood DC, Anderson SC, Wall M, Kardon RH (2007). Structure versus function in glaucoma: an application of a linear model. Invest Ophthalmol Vis Sci.

[13] Leung CK, Medeiros FA, Zangwill LM, Sample PA, Bowd C, Ng D, Cheung CY, Lam DS, Weinreb RN (2007). American Chinese glaucoma imaging study: a comparison of the optic disc and retinal nerve fiber layer in detecting glaucomatous damage. Invest Ophthalmol Vis Sci.

[14] Schlottmann PG, De Cilla S, Greenfield DS, Caprioli J, Garway-Heath DF (2004). Relationship between visual field sensitivity and retinal nerve fiber layer thickness as measured by scanning laser polarimetry. Invest Ophthalmol Vis Sci.

[15] Leung CK, Chong KK, Chan WM, Yiu CK, Tso MY, Woo J, Tsang MK, Tse KK, Yung WH (2005). Comparative study of retinal nerve fiber layer measurement by StratusOCT and GDx VCC II: structure/function regression analysis in glaucoma. Invest Ophthalmol Vis Sci.

[CR16] The pre-publication history for this paper can be accessed here:http://www.biomedcentral.com/1471-2415/14/118/prepub

